# Evaluation of Microbiological and Physicochemical Parameters of Alternative Source of Drinking Water: A Case Study of Nzhelele River, South Africa

**DOI:** 10.2174/1874285801812010018

**Published:** 2018-02-28

**Authors:** Joshua N. Edokpayi, John O. Odiyo, Elizabeth O. Popoola, Titus A.M. Msagati

**Affiliations:** 1 Department of Hydrology and Water Resources, University of Venda, Private Bag X5050, Thohoyandou, 0950, South Africa; 2 Department of Chemical Sciences, Yaba College of Technology, Nigeria; 3 College of Science, Engineering & Technology, Nanotechnology and Water Sustainability Research Unit, University of South Africa, South Africa

**Keywords:** *E. coli*, *Enterococci*, Public health, Water quality, Drinking water, Pollution

## Abstract

**Background:**

Access to clean and safe drinking water is still a problem in developing countries and more pronounced in rural areas. Due to erratic supply of potable, rural dwellers often seek for an alternative source of water to meet their basic water needs. The objective of this study is to monitor the microbiological and physicochemical water quality parameters of Nzhelele River which is a major alternative source of drinking water to villages along its course in Limpopo province of South Africa.

**Methods:**

Membrane filtration method was employed in evaluating the levels of *E. coli* and *Enterococci* in the river water from January-June, 2014. Specialized multimeter was used to measure the pH, electrical conductivity and turbidity of the river water. Ion Chromatograph was used to measure major anions such as fluoride, chloride, nitrate and sulphate in the water.

**Results:**

High levels of *E. coli* (1 x 10^2^ - 8 x 10^4^ cfu/100 mL) and *enterococci* (1 x 10^2^ – 5.7 x 10^3^ cfu/100 mL) were found in the river water and exceeded their permissible limits of 0 cfu/100 mL for drinking water. Turbidity values ranged from 1.12-739.9 NTU. The pH, electrical conductivity, chloride, fluoride, nitrate and sulphate levels were below their permissible limits for drinking water.

**Conclusion:**

The river water is contaminated with faecal organisms and is unfit for drinking purposes. However, the levels of the major anions accessed were within the permissible limits of drinking water.

## 1 INTRODUCTION

Clean and safe water is an important natural resource for the sustainability of life and a healthy economy. Freshwater availability is one of the major problems facing the world and approximately one third of drinking water requirement of the world is obtained from surface sources like rivers, dams, lakes and canals [1]. These sources of water also serve as best sinks for the discharge of domestic and industrial wastes [2, 3]. The biggest threat to sustainable water supply in South Africa is the contamination of available water resources through pollution [4]. About 43, 000 of South Africans might die annually as a result of diarrhoea diseases. Many communities in South Africa still rely on untreated or insufficiently treated water from surface resources such as rivers and lakes for their daily supply, and have no or limited access to adequate sanitation facilities thus at are a high risk of waterborne diseases [5]. Since 2000, there has been a dramatic increase in the episodes of waterborne diseases in South Africa [5, 6].

Nevondo and Cloete [7], reported that there are constraints in the provision of bulk supply of potable water to rural areas of South Africa despite the intervention of the government. Some of these constraints are largely due to insufficient allocation of funds and inadequate human resources. In communities where potable water is supplied, it is usually erratic and unreliable forcing residents to revert to surface water from rivers for their domestic needs [7, 8]. One of the major threats to public health by the use of such contaminated water is the presence of high concentration of pathogens capable of compromising the health of the people that drink and use the water for recreational and agricultural purposes [8].

Faecal contamination is one of the priority environmental problems associated with the use of surface water [9]. Diseases may be transmitted not only through the drinking of contaminated water, but also through skin contact during recreational activities or by eating raw crops irrigated with contaminated water [10]. Water resources can be directly contaminated by natural runoff after rainfall events, effluents from wastewater treatment facilities, agricultural and industrial effluents and several other anthropogenic activities [10, 11]. Since it is extremely difficult to test for each pathogenic organism present in water due to large diversity, low abundance of each species and the absence of standardized methods for their detection; regulatory authorities have resort to the use of indicator organisms [12-16].

Several indicator organisms and pathogens used to assess the microbial contamination of water include total coliforms count, faecal coliforms, faecal streptococci, coliphages, C. perfringens, Salmonella and heterotrophic plate counts [8, 17, 18]. The drawbacks of some of these methods for routine monitoring have further led to the preference of some over the others [19, 20]. *E. coli* is a widely accepted indicator organism for drinking water; although its use has been criticized by some scientists, it is still widely used for routine monitoring of domestic water [20, 21]. The United State Environmental Protection Agency (USEPA) has prescribed *Enterococci* and *E. coli* as the most appropriate indicator organisms for recreational water due to a study conducted on beaches in the United States of America [20]. It is therefore necessary to estimate the water quality of Nzhelele River which is used by residents of several communities surrounding it for domestic, recreational and agricultural purposes so as to prevent any episode of waterborne diseases.

### 1.1 The Study Area

Nzhelele River in Limpopo province (22^o^21’08” S and 30^o^22’19” E) is a major watercourse in Limpopo province of South Africa [22]. Nzhelele River have Mutamba, Tshiruru, Mufungudi, Mutshedzi and Wyllie Rivers as its tributaries. It joins the Limpopo River 33 km east of Musina. The river catchment area is 2, 436 km^2^ and have an average annual precipitation and evaporation of 422 mm and 2160 mm, respectively [23]. The region is semi-arid with seasonal rainfall events. Rainfall, temperature and humidity data of the river catchment were obtained from the South African Weather Service Table (**1**). Daily temperature in the catchment varies between 20–40^o^C (wet season) and 12–22^o^C (dry season), respectively [24, 25]. The region is characterized by a warm wet season which is associated with high temperatures up to 40°C usually between October and March (with peak precipitation in January and February) and cold dry season (April-September).

Upstream land use in the catchment area includes subsistence and commercial agriculture, schools, formal and informal human settlements, hospital, garages, waste stabilization ponds (WSPs), brick making factories, sand and gravel mining [26]. Brick making are usually done at the bank of the river. Siloam WSPs releases its hospital effluent directly into the river. Abstraction of water from the river for drinking and other domestic purposes without treatment is a common practice. Pipes are usually connected to the river by farmers for irrigation of their crops. Low scale fishing is done further downstream of the river. The Map of the study area is shown in Fig. (**1**). There is limited water infrastructure and high unemployment around villages in Nzhelele River catchment. The major source of drinking water for the people around the river catchment is potable water supplied from the Municipal Water Works, however, this supply is erratic and people are forced to look for alternative sources. Residents depend mainly on groundwater, springs and river water [27].

## 2 MATERIALS AND METHODS

### 2.1 Sampling

All sample bottles used were soaked in a livid detergent, followed by rinsing with tap water until they were free of detergent. Six samples were collected monthly from 30^th^ January to 10^th^ June, 2014, making a total of 36 samples. The samples were collected randomly along different sampling locations in Nzhelele River using sterile sampling bottles. Each sample was collected by submerging the sample container into the river at about 100–300 mm below the surface with an open end facing against the current flow direction [28]. Field measurements of pH and Electrical conductivity (EC) were performed using a 340i Multimeter (WTW, Weilheim, Germany) while Turbidity was measured with a Tobcon turbidimeter (TB200, Orbeco Hellige, Sarasota, FL, USA). The samples were transported on ice chest to microbiology laboratory of the University of Venda.

### 2.2 Analysis of Faecal Indicator Organisms

All samples were analysed within 6 hours of collection as recommended by the American Public Health Association (APHA) except for January samples which were analysed within 16 hours of collection this was because of difficulties to reach the sampling sites due to heavy rainfall encountered on the way before reaching them. Each water sample was analysed for *E. coli* and *Enterococci* levels. Samples (100 mL) were diluted (1:10) in accordance with level of pollution, and analysis was performed using the membrane filtration culture method in accordance with the standard methods described by APHA [29].

### 2.3 Analysis of Anions

The method reported by Edokpayi **et al.** [30] was employed in this study: The samples were filtered through 0.45-micron syringe filter and placed in an autosampler connected to Metrohm 850 Ion Chromatograph (IC) supplied by Metrohm, Switzerland. Calibration standards for fluoride, chloride, nitrate and sulphate were prepared from two multi element standards. 1 mg/L, 5 mg/L, 10 mg/L and 20 mg/L were prepared by serial dilution from a stock solution of 100 mg/L. The eluent used was a combination of Na_2_CO_3_ and NaHCO_3_; prepared by weighing accurately 0.168 g and 0.6784 g into 2 L volumetric flask and filled to the mark using ultrapure water. 0.5M sulfuric acid was used as a regenerant solution. Prior to analysis, the eluent was degassed using an ultrasonic bath for 15 minutes. The IC has a flow rate of 0.7 mL/min, maximum and minimum pressure of 15.0 mPa and 0.1 mPa respectively.

### 2.4 Validation of Analytical Methodology

In order to validate the analytical methodology, recovery studies were performed. Known concentrations of the test analyte were added to the sample. The concentrations of both the spiked and unspiked samples were determined and percentage recovery was obtained.

### 2.5 Statistical Analysis

Delta Graph 7 was used for drawing the graphs. SPSS version 20.0 was used for evaluating the average values obtained from each sampling month. The student t-test of SPSS was employed in the comparing of the means with probability set at p<0.05.

## 3 RESULTS

### 3.1 Faecal Indicator Organisms

**Fig. (2):**
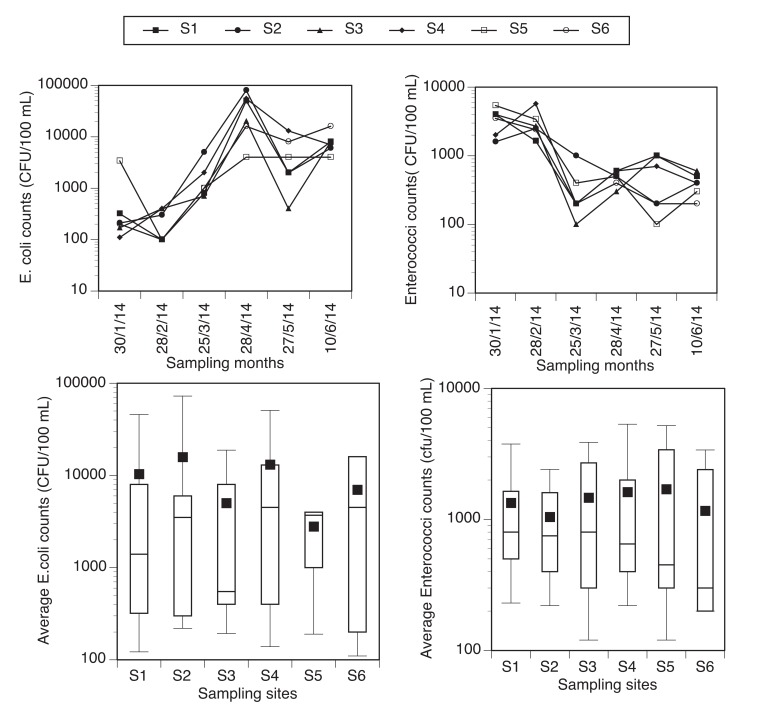
A logarithmic plot showing the total counts of *E. coli* (top left) and *Enterococci* (top right) and the box-and-whisker plots of *E. coli* (down left) and *Enterococci *(down right) in the sampling sites along Nzhelele River. The SANS and WHO permissible limit for faecal coliform (*E. coli* and *Enterococci*) in drinking water is 0 cfu/100 mL).

Total *E. coli* and *enterococci* counts in the samples from six different sites along the river are presented in Fig. (**2**) The box-and-whisker plots which indicate the mean (diamond) of the levels of *E. coli* and *enterococci* in each site, the first, second, and third quartiles (box), and minimum and maximum (whiskers) are also presented in (Fig. **2**).

### 3.2 Physicochemical parameters

The percentage recovery obtained for fluoride, chloride, nitrate and sulphate concentrations were 93%, 96%, 95% and 97%, respectively. The average pH values ranged between 7.21-7.76 Table (**2**). The average EC values varied from 83.47 -136.07 µS/cm, during the sampling period. Fig. (**3**) shows the turbidity values measured during the sampling periods.

Low levels of anions were found in the water samples collected from the various sampling sites Fig. (**4**). All the anions investigated complied with the SANS permissible limit of drinking water. Table **3** shows the level of correlation and significance between physicochemical and microbiological parameters in the wet and the dry season.

## 4 DISCUSSION

High levels of *E. coli* and e*nterococc*i were found in the river water which could pose a health risk to the consumers of this water resource. Higher *E. coli* levels were found in the dry season (April-June) than in the wet season (January-March) (Fig. **2**). A different trend was observed as higher levels of *enterococci* were recorded in the wet season. The results in the wet season is unexpected because of more incidence of rainfall which could lead to high surface runoff into the river, also owing to poor solid waste and wastewater collection systems within the river catchment. Retamozo *et al.* [33] have shown that bacteria counts are expected to be higher during rainy season and in turbid water which is not the case for *E. coli* in this study. The reason for this finding could also be due to high temperature during wet season which is characteristic of the study area than during dry season, thus providing a good temperature for the incubation of bacteria. During the wet season, most animals are restricted to farms since there is sufficient water for them collected from rainwater harvesting.

The high level of *E. coli* determined in the dry season could be due to high incidence of human and animal wastes in the water during the dry season which is so limited in the wet season. During the dry seasons, there is scarcity of water, and people including animals often resort to river waters. Edokpayi [34] reported that Siloam waste stabilization ponds discharges effluent with *E. coli* and *enterococci* levels in the range of 2 x 10^3^- 7.7 x 10^5^ cfu/100 mL and 2 x 10^3^-7 x 10^4^ cfu/100 mL, respectively into Nzhelele River. The contribution of this point source and other non-point sources definitely contributes to the high levels of faecal contamination determined in Nzhelele River.

*Enterococci* counts were higher as expected in the wet season than in the dry season January-March) (Fig. **2**). The highest level was observed in January in the wet season (3.42 x 10^3^ cfu/100 mL) and the lowest counts in June (4 x 10^2^ cfu/100 mL) in the dry season. The average counts of *E. coli* were higher than *enterococci* count but there was negative correlation between the levels of both indicator organisms.

Several trends have been reported for bacteria levels in surface water; some of which are higher in the wet season and others in the dry season [25, 35-38]. There are several environmental drivers that influence the levels of bacteria in water. Generally, high sediment load from land into surface water bodies due to rainfall events usually lead to high counts of faecal indicator organisms. Temperature is another factor that could influence the levels of pathogenic organisms in surface water. Bacteria grow faster at higher temperature than at lower temperature. High levels of nutrient also influence the growth rate of bacteria. Levy *et al.* [40] reported that bacterial levels in water are due to a complex interaction of various effects in varying conditions at different times.

A higher count of faecal indicator organism was reported by Sibanda *et al.* [39] in Dryini sampling point during the dry season than in the wet season in their studies on Tyume River in Eastern Cape Province in South Africa which was opposite to the findings of Fatoki *et al.* [40] in Umtata River catchment. The results obtained indicate that water from Nzhelele River is unfit for domestic use as it exceeded the SANS and WHO permissible limit of 0 cfu/100 mL [31, 32]. The river water is also not suitable for recreational and agricultural water use as it exceeds the permissible limit of the South African Department of Water and Forestry guidelines (≤130 cfu/100 mL and ≤1 cfu/100 mL, respectively) for such uses [41].

The presence of suspended substances in water like clays, silts and micro-organisms causes a cloudy appearance of water. The values obtained for turbidity measurements varied greatly as expected from January to June. Very high turbidity values were obtained from January-March which is the wet season of the study area than from April-June (dry season). This could be due to more incidence of rainfall in the wet season. In the upstream of Nzhelele River, there is sand mining, bricks making industries at the bank of the river and commercial faming, therefore, runoff during rainfall events can lead to increased sedimentation of the river thus contributing to high turbidity values determined in the wet season. The mean values obtained for both seasons were higher than SANS and WHO permissible limit of ≤1 NTU for domestic water use [31, 32] Fig. (**3**). The average turbidity values varied significantly for both the wet and the dry seasons (p<0.05). Rainfall data in the catchment area varied between 0.5 mm and 258.82 mm Table (**1**). Highest precipitation was observed in January and the lowest in June. There was a slight change in the precipitation pattern of the study area. January and February are usually the months with highest precipitation in the river catchment but in this study, the highest precipitation was recorded in January and March. The average monthly temperature however decreased from 24.1^o^C in January to 17.5^o^C in June. The pH and conductivity levels obtained in this study complied to various regulatory standards for drinking water. Low EC values were obtained upstream of the rivers while higher values were obtained downstream due to anthropogenic activities. Higher EC values was measured and computed for the dry season than in the wet season (Table **2**). Low EC could be due to dilution effect as a result of more volume of water in the river due to increased precipitation. However, in the dry season, dilution effect is cancelled out, this coupled with evaporation can lead to increased levels of dissolved ions concentration in the river water.

Correlation studies on the data obtained were carried out. The levels of *E. coli* correlated negatively and non-significantly (r = -0.272, p>0.05) with the levels of *enterococci*. Positive and significant correlation was obtained between the levels of *enterococci* and turbidity (0.686, p<0.01) and between nitrate concentration and pH (r = 0.493, p<0.01). Other correlational relationship exhibited between the various parameters studied in Nzhelele River is shown in Table (**4**). The mean difference in* enterococci* and *E. coli* counts in the river did not differ significantly (p>0.05). Significant difference in the means of turbidity and the faecal indicator organisms were obtained (P<0.01).

The concentrations of the anions determined did not present any health risk to the health of the consumers of this resource. Although high concentration of fluoride exceeding 1.5 mg/L can affect the bones and teeth of humans, the average levels observed in this study was lower than 1 mg/L for each sampling month. Nitrogen is an important plant nutrient and often applied to agricultural lands to enhance plants productivity. Although various forms of agriculture are practiced in the study area, the concentrations of nitrates determined complied to SANS guidelines for safe drinking water [31].

## CONCLUSION

Surface water should be protected against undue anthropogenic influence. The river examined in this study and many others which are used by rural dwellers around the world are increasingly loaded with various classes of pollutants from both point and non-point sources. Although the physicochemical water quality parameters investigated in this study complied with the benchmark values, the water is polluted with faecal matter with potential risk of waterborne diseases to the users of this resource. In order to prevent negative episode of waterborne diseases, it is recommended that water abstraction of water from Nzhelele River for domestic purposes without proper treatment should be discouraged. Cheap and efficient point of use water treatment devices should be developed.

## Figures and Tables

**Fig. (1):**
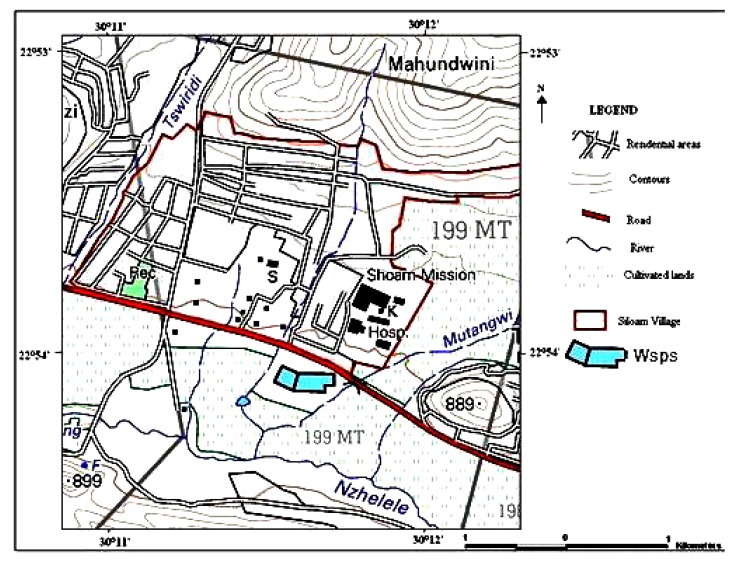
Map of the study area [27].

**Fig. (3):**
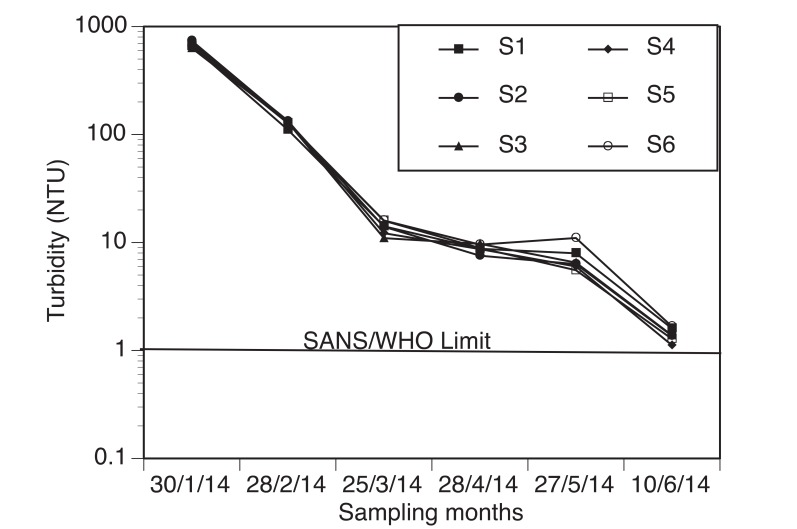
Turbidity levels of water samples collected from January-June, 2014. The SANS operational and aesthetic permissible limit of turbidity for drinking water is 1 and 5 NTU, respectively.

**Fig. (4):**
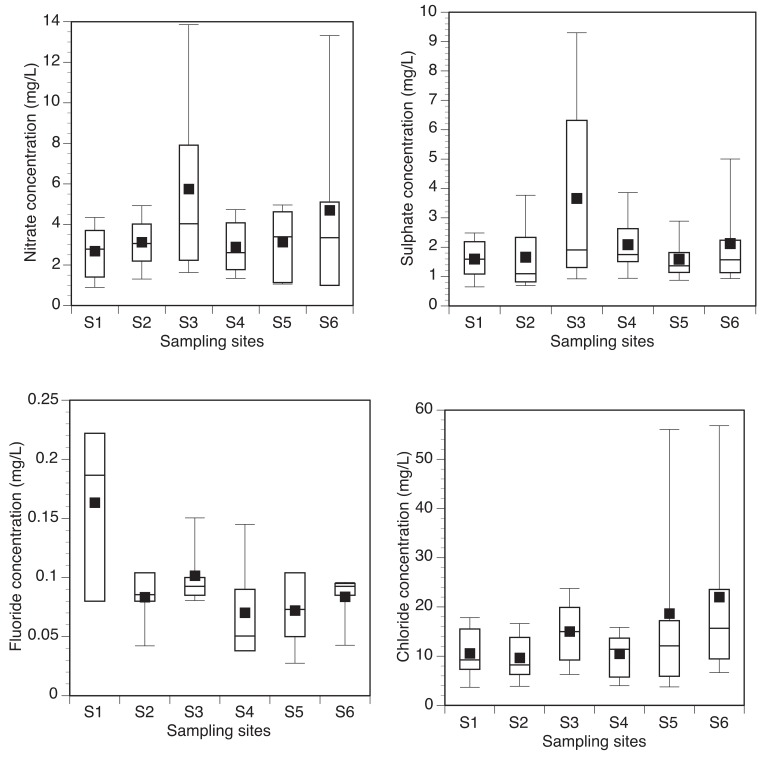
Box-and-whisker plots showing nitrate (top left), sulphate (top right), fluoride (down left) and chloride (down right) concentrations in the various sampling sites of Nzhelele River. SANS permissible limit of nitrate, sulphate, chloride and fluoride in drinking water are >40 mg/L, 250 mg/L, 300 mg/L and 1.5 mg/L, respectively.

**Table 1:** Climate data of the river catchment.

**Months**	Total Rainfall (mm)	Average Temperature (^o^C)	Average Humidity (%)
January	258.82	24.1	69.3
February	90.94	23.2	72.7
March	257.29	22.8	73.9
April	11.92	20.7	63.4
May	8.63	18.8	58.7
June	0.5	17.5	48.3

Where Avg is average, min is minimum, max is maximum, Temp is temperature and Hum is humidity.

**Table 2:** Average level of physicochemical parameter analysis.

**Months**	**pH**	**EC (µS/cm)**
January	7.76±0.25	83.47±8.92
February	7.44±0.45	101.45±37
March	7.67±0.05	97.93±11.4
April	7.21±0.13	120.68±2.3
May	7.54±0.07	130.07±16.16
June	7.67±0.06	136.07±3.35
SANS guideline [31]	5-9.7	1700
WHO guideline [32]	6.5-8.5	6000

**Table 3:** Results from correlation and statistical mean difference between physicochemical and microbiological parameters of Nzhelele Rivers in the wet and the dry season.

Parameters	Correlation Value in Nzhelele River	p-Value in Nzhelele River
pH (wet and dry)	-0.475	0.096
EC (wet and dry)	0.210	0.012*
Turbidity (wet and dry)	0.723**	0.001**
F^-^ (wet and dry)	0.062	0.408
Cl^-^ (wet and dry)	0.639**	0.001**
NO_3_^-^ (wet and dry)	0.492*	0.04*
SO_4_^2-^ (wet and dry)	-0.015	0.746
*E. Coli* (wet and dry)	-0.269	0.008**
*Enterococci *(wet and dry)	-0.13	0.001**

**mean difference and correlation is significant at the 0.01 level (2-tailed), * mean difference and correlation is significant at the 0.05 level (2-tailed). The p-value for significant correlation is not presented in this table.

**Table 4:** Results from the correlation studies on the various parameters investigated in Nzhelele River.

	*E. Coli*	*Ent*	pH	EC	T	Cl^-^	NO_3_^-^	F^-^	SO_4_^2-^
*E. Coli*	1	-.272	-.410^*^	-.147	-.258	-.184	-.264	-.048	-.071
Ent		1	.191	.245	.686^**^	-.317	-.034	.249	-.299
pH			1	.137	.388^*^	.210	.149	.303	.057
EC				1	.263	-.086	-.184	.082	-.374^*^
T					1	-.435^**^	-.121	.297	-.227
Cl^-^						1	.493^**^	.257	.179
NO_3_^-^							1	.245	.270
F^-^								1	-.083
SO_4_^2-^									1

EC is electrical conductivity, T is turbidity, *En*t is enterococci; *correlation is significant at 0.05 level (2-tailed); ** correlation is significant at 0.01 level (2-tailed).
