# Supplementary material for: Evaluation of Microbiological and Physicochemical Parameters of Alternative Source of Drinking Water: A Case Study of Nzhelele River, South Africa
Source: Open Microbiol J. 2018 Feb 28;12:18–27. doi: 10.2174/1874285801812010018 (PMC5842400; doi:10.2174/1874285801812010018)
Supplement: Supplementary file 1 [file TOMICROJ-12-18_SD1.pdf]

Send Orders for Reprints to reprints@benthamscience.ae

The Open Microbiology Journal, 2018, *12*, i i

BENTHAM OPEN

CrossMark

The Open Microbiology Journal

Supplementary Material

Content list available at: www.benthamopen.com/TOMICROJ/

DOI: 10.2174/1874285801812010018

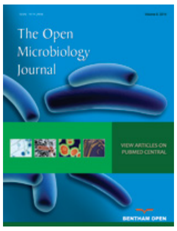

Evaluation of Microbiological and Physicochemical Parameters of Alternative Source of Drinking Water: A Case Study of Nzhelele River, South Africa

Joshua N. Edokpayi[*,1], John O. Odiyo[1], Elizabeth O. Popoola[2] and Titus A.M. Msagati[3]

[1]*Department of Hydrology and Water Resources, University of Venda, Private Bag X5050, Thohoyandou, 0950, South Africa*
[2]*Department of Chemical Sciences, Yaba College of Technology, Nigeria*
[3]*College of Science, Engineering & Technology, Nanotechnology and Water Sustainability Research Unit, University of South Africa, South Africa*

Received: November 21, 2017 Revised: January 11, 2018 Accepted: January 17, 2018

Supplementary data 1. Average level of physicochemical parameter analysis.

Months	pH	EC (µS/cm)	Turbidity (NTU)
January	7.76±0.25	83.47±8.92	678±38
February	7.44±0.45	101.45±37	126±8
March	7.67±0.05	97.93±11.4	13.87±2
April	7.21±0.13	120.68±2.3	8.86±0.77
May	7.54±0.07	130.07±16.16	7.22±2.03
June	7.67±0.06	136.07±3.35	1.40±0.2
DWAF guidelines	6.5-8.5	<700	<1
WHO guidelines	6.5-8.5	6000	<1

© 2018 Edokpayi *et al*.

This is an open access article distributed under the terms of the Creative Commons Attribution 4.0 International Public License (CC-BY 4.0), a copy of which is available at: (https://creativecommons.org/licenses/by/4.0/legalcode). This license permits unrestricted use, distribution, and reproduction in any medium, provided the original author and source are credited.
